# Distinct Gut Microbial Signature and Host Genetic Variants in Association with Liver Fibrosis Severity in Patients with MASLD

**DOI:** 10.3390/nu16121800

**Published:** 2024-06-07

**Authors:** Nantawat Satthawiwat, Thananya Jinato, Sawannee Sutheeworapong, Natthaporn Tanpowpong, Natthaya Chuaypen, Pisit Tangkijvanich

**Affiliations:** 1 Center of Excellence in Hepatitis and Liver Cancer, Department of Biochemistry, Faculty of Medicine, Chulalongkorn University, Bangkok 10330, Thailand; 6371007030@student.chula.ac.th (N.S.); ji.thananya@gmail.com (T.J.); natthaya.ch56@gmail.com (N.C.); 2 Doctor of Philosophy Program in Medical Biochemistry, Department of Biochemistry, Faculty of Medicine, Chulalongkorn University, Bangkok 10330, Thailand; 3 Systems Biology and Bioinformatics Research Unit, Pilot Plant Development and Training Institute, King Mongkut’s University of Technology Thonburi, Bangkok 10150, Thailand; sawannee.sut@kmutt.ac.th; 4 Department of Radiology, Faculty of Medicine, Chulalongkorn University, Bangkok 10330, Thailand; tanpowpongn@gmail.com

**Keywords:** metabolic dysfunction-associated steatotic liver disease (MASLD), fibrosis, magnetic resonance elastography (MRE), gut microbiota

## Abstract

Gut microbiota might affect the severity and progression of metabolic dysfunction-associated steatotic liver disease (MASLD). We aimed to characterize gut dysbiosis and clinical parameters regarding fibrosis stages assessed by magnetic resonance elastography. This study included 156 patients with MASLD, stratified into no/mild fibrosis (F0–F1) and moderate/severe fibrosis (F2–F4). Fecal specimens were sequenced targeting the V4 region of the 16S rRNA gene and analyzed using bioinformatics. The genotyping of *PNPLA3*, *TM6SF2*, and *HSD17B13* was assessed by allelic discrimination assays. Our data showed that gut microbial profiles between groups significantly differed in beta-diversity but not in alpha-diversity indices. Enriched *Fusobacterium* and *Escherichia_Shigella*, and depleted *Lachnospira* were found in the F2–F4 group versus the F0–F1 group. Compared to F0–F1, the F2–F4 group had elevated plasma surrogate markers of gut epithelial permeability and bacterial translocation. The bacterial genera, *PNPLA3* polymorphisms, old age, and diabetes were independently associated with advanced fibrosis in multivariable analyses. Using the Random Forest classifier, the gut microbial signature of three genera could differentiate the groups with high diagnostic accuracy (AUC of 0.93). These results indicated that the imbalance of enriched pathogenic genera and decreased beneficial bacteria, in association with several clinical and genetic factors, were potential contributors to the pathogenesis and progression of MASLD.

## 1. Introduction

Metabolic dysfunction-associated steatotic liver disease (MASLD) has increasingly become the most common chronic liver disease worldwide, affecting approximately one-third of the global population [1]. This new definition of non-alcoholic fatty liver disease (NAFLD) depends on a combination of hepatic steatosis and the clinical phenotype of metabolic dysfunction [1]. MASLD can lead to chronic inflammation (steatohepatitis), progressive hepatic fibrosis, and hepatocellular carcinoma (HCC) [2]. Accumulating evidence also indicates that MASLD is associated with increased risks of extrahepatic manifestations, namely cardiovascular disease (CVD), chronic kidney disease (CKD), and cancers [3]. MASLD is strongly related to obesity; however, approximately 20% of patients have a normal body mass index (BMI < 23 kg/m^2^ in Asian populations and <25 kg/m^2^ in non-Asian populations) [4]. Despite its high prevalence, the clinical management of MASLD is still difficult due to the lack of approved pharmacological treatments. Thus, lifestyle modifications, including diet, exercise, and weight reduction, remain the recommended approaches [5]. As approximately 20–30% of patients will finally develop progressive liver disease, it is essential to assess the disease severity and early identification of individuals at high risk of advanced MASLD. Although liver biopsy is considered the gold standard for the diagnosis of steatosis and fibrosis, this procedure has a risk of complications and sampling errors due to the uneven distribution of histopathology [6]. Alternatively, magnetic resonance elastography (MRE) is now considered the most reliable non-invasive method for assessing liver fibrosis in patients with MASLD [7].

The pathophysiology of MASLD is complex and mediated by several risk factors, including lifestyle behavior, environmental factors, and metabolic disorders such as type 2 diabetes (T2DM), obesity, and insulin resistance [8]. Moreover, genetic susceptibility has been shown to play a role in the pathogenesis of MASLD. These genetic variants include single nucleotide polymorphisms (SNPs) in the *patatin-like phospholipase domain containing 3 (PNPLA3)*, *transmembrane 6 superfamily member 2 (TM6SF2)*, and *17β-hydroxysteroid dehydrogenase 13 (HSD17B13)* [9]. Additionally, recent data indicate the essential role of gut microbiota as a mechanistic factor in the development and progression of MASLD via the gut–liver axis [10,11]. Specifically, alterations in gut microbial composition with an increase in potentially harmful and a decline in the abundance of beneficial bacterial genera, as well as a decreased bacterial diversity, have been recognized as contributing factors associated with progressive MASLD. Moreover, the translocation of bacteria and their metabolites, accompanied by increased gut permeability or so-called “leaky gut syndrome”, could also modulate the pathogenesis of MASLD [12]. It has been shown that the heterogeneity of gut microbial composition can be modulated by different dietary patterns [13]. However, current data linking gut microbiota to disease severity of patients with MASLD when considering other clinical parameters, including host genetic variants and dietary patterns, remain limited.

In this study, we used MRE to assess the severity of fibrosis and magnetic resonance imaging-proton density fat fraction (MRI-PDFF) for evaluating steatosis in a well-documented clinical cohort of Thai patients with MASLD. Our study aimed to characterize gut dysbiosis and various clinical characteristics that might impact the severity of fibrosis. In this respect, we analyzed the relationship among several factors using logistic regression analyses to control potential confounding effects. Taking several variables into account, our results could provide integrative information toward better clinical decision-making for patients with MASLD.

## 2. Materials and Methods

### 2.1. Participants and Study Design

A total of 156 Thai patients with MASLD were prospectively enrolled in this cross-sectional cohort between 2022 and 2023 in the outpatient liver clinic at the King Chulalongkorn Memorial Hospital, Thailand. Written informed consent was obtained from each participant, and the study was approved by the Institute Ethics Committee (IRB No. 981-64). The study was conducted following the Declaration of Helsinki and the principles of Good Clinical Practice.

The inclusion criteria were individuals aged ≥ 18 years with the diagnosis of liver steatosis based on MRI-PDFF grade ≥ 1 (defined as MRI-PDFF ≥ 5.4%) [14]. Exclusion criteria were (1) concomitant chronic viral hepatitis, autoimmune hepatitis, and other chronic liver diseases; (2) presence of other disorders causing secondary steatosis such as human immunodeficiency virus (HIV) infection; (3) presence of cirrhotic complications (e.g., ascites and variceal bleeding), (4) known malignancies including HCC, and (5) self-reported daily alcohol consumption of >10 g in women and >20 g in men. All participants were advised not to take any herbal or nutritional supplements, probiotics and prebiotics, antibiotics, and proton pump inhibitors at least 4 weeks before enrollment. The clinical data of patients with MASLD at the enrollment were recorded. Anthropometric measurements were performed by research assistant nurses.

### 2.2. Assessment of Liver Stiffness and Steatosis

MRE and MRI-PDFF were performed to assess liver stiffness and steatosis, respectively, by the MR imaging system Philips Ingenia at 3.0 T (Philips Healthcare, Best, the Netherlands). Based on a systematic review and meta-analysis of MASLD, the cut-off values for fibrosis ≥ F1 ≥ F2, ≥ F3, and F4 obtained by MRE measurement were 2.6, 3.0, 3.6, and 4.7 kPa, respectively [7]. For MRI-PDFF, the cut-off values for diagnosing steatosis grades ≥ 1, ≥2, and ≥3 were 5.4%, 15.4%, and 20.4% respectively [14]. Acquired imaging data were interpreted by a radiologist who was blinded to the clinical and laboratory information of the patients.

### 2.3. Fecal Collection, DNA Extraction and Sequencing

Participants in the study were advised to collect approximately 1 g of feces in DNA/RNA Shield Fecal Collection Tubes (Zymo Research, Irvine, CA, USA). The fecal samples were then vigorously shaken and stored at −80 °C until they were further processed. DNA from the fecal samples was extracted using the ZymoBIOMICS DNA Miniprep Kit (Zymo Research, USA), following the manufacturer’s instructions. The concentration and purity of total DNA were assessed using a DeNovix™ UV–Vis spectrophotometer, and the DNA was preserved at −20 °C until further experiments. Subsequently, for amplicon-based 16S rRNA gene sequencing, the V4 hypervariable regions were amplified using the forward primer 515F (5′-GTGCCAGCMGCCGCGGTAA-3′) and the reverse primer 806R (5′-GGACTACHVGGGTWTCTAAT-3′). This procedure was followed by paired-end sequencing on the Illumina MiSeq 2 × 300 bp platform (San Diego, CA, USA) conducted at Mod Gut Co., Ltd. (Bangkok, Thailand).

### 2.4. Data Processing and Analysis

The raw sequencing data from each sample, obtained via the Illumina platform, were processed using the nfcore/ampliseq analysis pipeline (https://zenodo.org/records/6411790, accessed on 6 March 2024). The procedure involved initially merging the paired-end reads to reconstruct the V4 region of the 16S rRNA gene by aligning overlapping sequences and eliminating chimeras. Following this process, the sequences were organized into unique sequence features based on the amplicon sequence variant (ASV) technique, leading to the generation of a table that captures the feature abundance across the samples with DADA2 [15]. Additionally, the microbial features were classified taxonomically with the SILVA 138.1 database. Subsequent analyses, including the examination of relative abundance, alpha-diversity, and beta-diversity indices, were conducted on the MicrobiomeAnalyst web platform (https://www.microbiomeanalyst.ca/, accessed on 6 March 2024). Differential bacterial abundance between groups was identified by linear discriminant analysis (LDA) effect size (LEfSe). Predictive pathway analysis was performed using MetaCyc Metabolic Pathway databases through PICRUSt2 [16].

### 2.5. Gut Microbiota Enterotypes and Dietary Patterns

The classification of gut microbiota profiles at the genus level into distinct enterotypes was conducted using Biotyper, an R software package (Version 4.2.3) based on the methodology as previously described [17,18]. The “biotyper.data.frame” function from the Biotyper package was employed to categorize the data into 4 distinct clusters. Subsequently, the enterotype for each cluster was identified by examining the top three genera within each cluster. Gut enterotypes and their dietary pattern correlations were determined by previous reference studies [17,18]. The cluster enriched in *Bacteroides* has been revealed to be associated with a meat-based diet, *Prevotella* associated with a carbohydrate-based diet, and *Firmicutes* with a vegetable-based diet.

### 2.6. Random Forest (RF) Classification

The Random Forest (RF) classifier was developed with the sklearn library in Python. Within receiver operating characteristic (ROC) curves, the F0–F1 group was labeled as class zero, and the F2–F4 group as class one. To perform Random Forest classification in our cohort, a 75:25 train/test split was used to evaluate the sensitivity and specificity across all possible thresholds. The results from those random classifiers were compared using a threshold value of 0.5 as a baseline.

### 2.7. Analysis of Microbial Surrogate Biomarkers and Inflammatory Cytokines

Peripheral blood samples from the patients were collected and handled within two hours to separate plasma, which was then preserved at −80 °C for downstream analysis. The levels of plasma lipopolysaccharide-binding protein (LBP) and intestinal fatty acid binding protein (I-FABP) were measured using ELISA kits from Hycult Biotech (Uden, The Netherlands). The samples were diluted at a ratio of 1:1000 for LBP and 1:2 for I-FABP, following the manufacturer’s recommended guidelines.

Serum interleukin-6 (IL-6) and tumor necrosis factor-α (TNF-α) levels were measured by flow cytometry using LEGENDplex Human CD8/NK Panel V02 (Biolegend, San Diego, CA, USA) according to the manufacturer’s instructions. Briefly, the antibody-conjugated bead against IL-6 and TNF-α was applied, followed by washing of the capture bead complex. Subsequently, a cocktail of biotinylated detection antibodies was introduced. Streptavidin-phycoerythrin was then added, and the resulting fluorescent intensity signal was measured using the BD FACSAria™ Fusion Cell Sorter (BD Biosciences, San Jose, CA, USA) and analyzed through the LEGENDplexTM Data Analysis Software (Version 15 February 2023).

### 2.8. DNA Extraction and the SNP Genotyping

DNA was extracted from peripheral blood mononuclear cells (PBMCs) using the phenol-chloroform isoamyl alcohol method. The quantity and quality of the DNA were assessed using a DeNovix™ UV–Vis spectrophotometer, and the samples were stored at −80 °C until further analysis. The genotyping of the *PNPLA3* rs738409, *TM6SF2* rs58542926, and *HSD17B13* rs6834314 genes was performed using allelic discrimination with TaqMan Probe Real-Time PCR Assays (ThermoFisher Scientific, Waltham, MA, USA), and fluorescent signals (FAM and VIC) were utilized for the detection according to previously described methods [19]. To ensure the accuracy of data interpretation, both positive and negative controls were included in each experiment. The allelic discrimination plot was analyzed using the QuantStudio™ 3 Real-Time PCR System (ThermoFisher Scientific, USA).

### 2.9. Statistical Analysis

The statistical evaluation of variables was performed by using SPSS (version 22.0.0, SPSS Inc., Chicago, IL, USA) and GraphPad Prism (version 9.5.0, Boston, MA, USA). Categorical data were analyzed using the Chi-square test and one-way ANOVA. The Mann–Whitney test was applied for comparisons between non-paired groups. Hardy–Weinberg equilibrium (HWE) was determined using Pearson’s Chi-square calculated using online software. Correlations between parameters were analyzed by Spearman’s rank test. Univariable and multivariable analyses were performed on SPSS using binary logistic regression to determine the parameters associated with F2–F4 fibrosis. A *p*-value of less than 0.05 was considered as statistically significant.

## 3. Results

### 3.1. Clinical Parameters of Patients

The clinical characteristics of 156 patients with MASLD according to fibrosis stages [no/mild fibrosis (F0–F1, n = 131) and moderate/severe fibrosis (F2–F4, n = 25)] are summarized in Table 1. Compared to patients with F0–F1, the F2–F4 group were significantly older and had a higher prevalence of T2DM and hypertension (HT). However, there was no difference in gender distribution, BMI, and the presence of dyslipidemia (DLP), as well as the serum levels of total cholesterol, HDL-cholesterol, LDL-cholesterol, and triglyceride between the groups. In addition, patients in the F2–F4 group had lower platelet counts but higher serum aspartate aminotransaminase (AST), alanine aminotransaminase (ALT), and average values of MRE measurement compared to those in the F0–F1 group. There was no significant difference between groups in terms of other clinical parameters, or MRI-PDFF values. In addition, there was a positive correlation between MRI-PDFF and serum triglyceride (r = 0.277, *p* = 0.001), but a negative correlation between MRI-PDFF and HDL level (r = −0.289, *p* = 0.001).

As the difference between females and males became increasingly crucial, we further analyzed the clinical characteristics of the patients according to gender (Supplementary Table S1). Overall, the baseline parameters regarding the severity of fibrosis in female and male individuals demonstrated similar trends compared to the studied population as a whole. Among female patients, however, the F0–F1 vs. F2–F4 groups had a comparable mean age and prevalence of HT. Among male subjects, there was a significant difference in MRI-PDFF value when compared between the F0–F1 and F2–F4 groups.

### 3.2. The Alpha and Beta Diversities of Gut Microbiota

**Figure 1:**
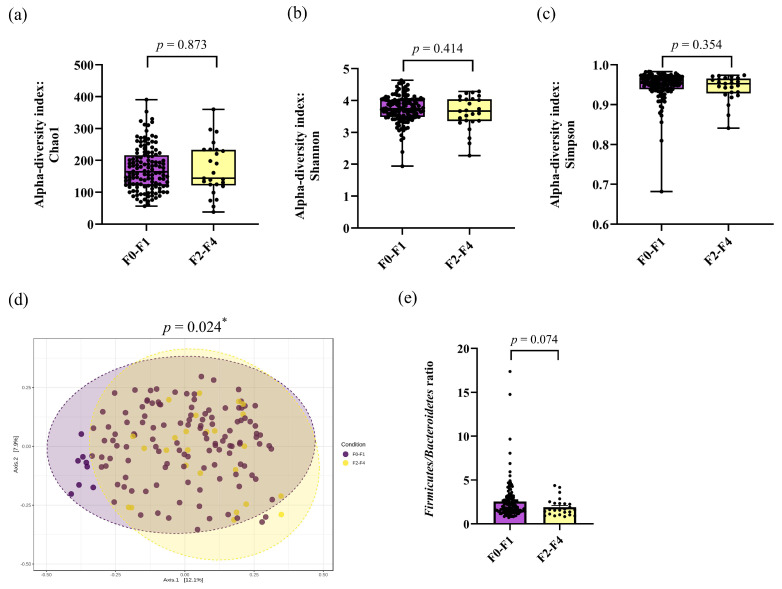
Gut microbial composition between the F0–F1 and F2–F4 groups. Alpha diversity: (**a**) Chao1 index, (**b**) Shannon index, (**c**) Simpson index, (**d**) beta diversity using principal coordinate analysis (PCoA) based on Bray–Curtis index between groups. Statistical significance was determined by pairwise PERMANOVA. (**e**) The *Firmicutes/Bacteroidetes* ratio. * *p*-value < 0.05.

To investigate the alpha diversity of bacteria, Chao1, Shannon, and Simpson indices were compared between patients with F0–F1 and F2–F4 fibrosis. The results showed no significant differences in any of the indices between the two groups (*p* = 0.873, *p* = 0.414, and *p* = 0.354, respectively). These data suggested that bacterial richness and evenness were not significantly different between no/mild fibrosis and moderate/severe fibrosis stages (Figure 1a–c).

To measure the compositional dissimilarity of bacteria between patients in the F0–F1 and F2–F4 groups, the Bray–Curtis dissimilarity index was assessed using permutational multivariate analysis of variance (PERMANOVA) tests. The data were visualized in a principal coordinate analysis (PCoA) plot, as shown in Figure 1d. The findings revealed a significant distinction in beta diversity between the F0–F1 and F2–F4 groups (PERMANOVA, *p* = 0.024).

### 3.3. Alteration of Taxonomic Level of Gut Microbiota and LEfSe Analysis

To determine whether the difference in gut microbial composition was associated with fibrosis severity, we compared the most relative abundance of taxa according to the fibrosis groups. Among the dominant phyla, the abundances of *Firmicutes* and *Bacteroidetes* between the F0–F1 and F2–F4 groups were 59.46% vs. 53.12% (*p* = 0.003) and 28.29% vs. 30.20% (*p* = 0.355), respectively. As a result, the *Firmicutes/Bacteroidetes* (F/B) ratio decreased in the F2–F4 group in comparison with the F0–F1 group, although a significant difference was not achieved (1.9 ± 1.0 vs. 2.6 ± 2.2, *p* = 0.074) (Figure 1e).

**Figure 2:**
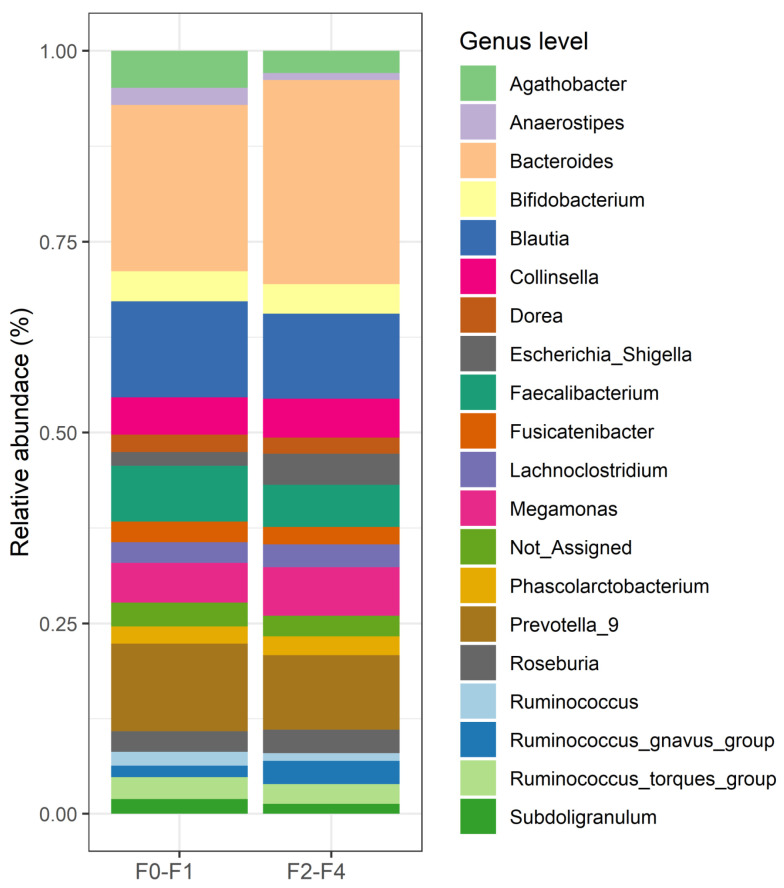
The top 20 relative bacterial compositions at the genus level in the F0–F1 and F2–F4 groups.

The top 20 relative abundances of bacteria at the genus level are shown in Figure 2. The relative abundances according to the patient groups were described as medians [IQR] as follows: *Bacteroides* [15.01% (8.91–23.83%)], *Blautia* [8.09% (6.46–11.08%)], *Faecalibacterium* [4.96% (2.19–8.23%)], *Agathobacter* [2.55% (0.47–5.16%)], *Collinsella* [2.31% (0.83–5.46%)], *Roseburia* [1.56% (0.53–3.00%)], Lachnoclostridium [1.54% (0.93–2.63%)], *Ruminococcus_torques_group* [1.45% (0.52–3.15%)], *Fusicatenibacter* [1.43% (0.57–2.88%)], *Dorea* [1.40% (0.93–2.26%)], *Bifidobacterium* [1.22% (0.30–3.16%)], *Eubacterium_hallii_group* [1.21% (0.69–1.70%)]; *Subdoligranulum* [1.06% (0.23–2.06%)]. Other bacteria, each constituting less than 1%, included *Sutterella*, *Anaerostipes*, *Parabacteroides*, *Butyricicoccus*, *Streptococcus*, *Lachnospiraceae_NK4A136_group*, and *Monoglobus*. Additionally, the top 50 relative abundances are available in Figure S1 (Supplementary Materials).

**Figure 3:**
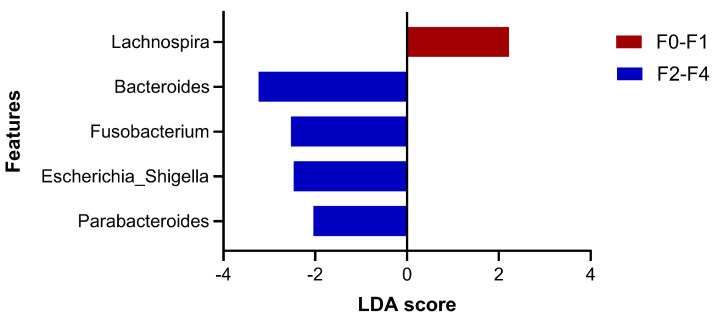
Linear discriminant analysis (LDA) effect size (LEfSe) analysis of gut microbiota between the two groups at the genus level (LAD > 2, *p*-value < 0.05).

To evaluate the most significant variations in microbiota compositions between the F0–F1 and F2–F4 groups, the LEfSe method was analyzed (Figure 3). The results showed the discriminating bacterial abundance in both groups as enriched *Lachnospira* was observed in the F0–F1 group, while four genera, including *Parabacteroides*, *Escherichia_Shigella*, *Fusobacterium*, and *Bacteroides* were increased in the F2–F4 group. Of note, the relative abundance of *Fusobacterium* was positively correlated with MRE value (r = 0.216, *p* = 0.007), while the abundance of *Lachnospira* was negatively correlated with MRE (r = −0.209, *p* = 0.009).

### 3.4. Discriminant Analysis and Predict Functional Pathways

**Figure 4:**
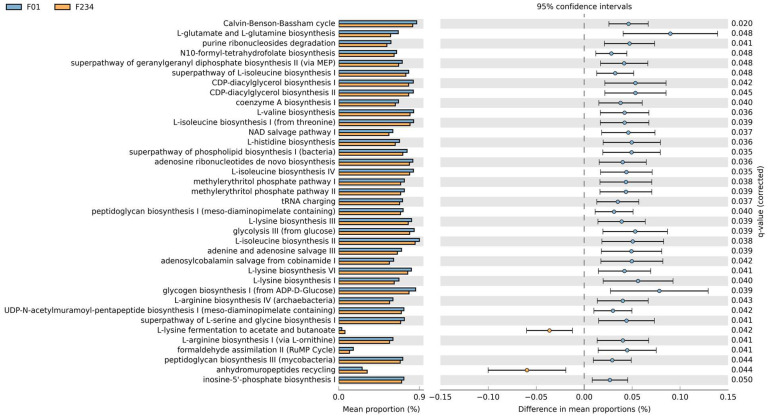
Functional pathways obtained with PICRUSt2 based on the MetaCyc Metabolic Pathway Database with a false discovery rate (FDR) <  0.05.

To predict the abundance of metabolic pathways (MetaCyc pathways) from differential bacteria in the F0–F1 versus F2–F4 groups, PICRUSt2 analysis was performed. In this respect, a total of 37 pathways were found across the sample groups. The significant differential pathways were obtained based on a false discovery rate (FDR) < 0.05 (Figure 4). For instance, L-lysine fermentation to acetate and butanoate and anhydromuropeptide recycling pathways were enriched in patients with the F2–F4 group. In contrast, other pathways, such as L-glutamate and L-glutamine biosynthesis, glycogen biosynthesis I (from ADP-D-Glucose), and glycolysis III (from glucose), were enriched in patients with F0–F1 fibrosis.

### 3.5. Dietary Patterns Associated with Fibrosis Severity and Microbiota Enterotypes

To identify the association between dietary patterns and fibrosis severity, Chi-square analysis was performed. The results indicated that nutritional enterotypes, including vegetable-based, meat-based, and carbohydrate-based diets, were not associated with the stage of fibrosis. (Pearson Chi-square, *p* = 0.617). However, the five microbial signatures from the LEfSe analysis showed different relative abundances among diverse groups of dietary patterns. Specifically, the relative abundance of *Bacteroides* was significantly increased in the meat-based diet compared to other dietary patterns. *Escherichia_Shigella* exhibited the lowest relative abundance in a vegetable-based diet. For *Fusobacterium*, the relative abundance was remarkably increased in the meat-based diet compared to the other dietary patterns. Moreover, the relative abundance of *Lachnospira* trended to increase in a vegetable-based diet and the level of *Parabacteroides* was decreased in a carbohydrate-based diet (Figure S2, Supplementary Materials).

### 3.6. Circulating Levels of Microbial Surrogates and Inflammatory Cytokines

To compare gut epithelial permeability and bacterial translocation between groups, plasma I-FABP and LBP levels were measured, respectively. Our data showed that the F2–F4 group had significantly higher I-FABP levels than the F0–F1 group [821.3 (600.9–1591.6 ng/mL) vs. 386.6 (144.9–812.5 ng/mL), *p* = 0.019]. Additionally, there was a significant increase in LBP levels in patients with F2–F4 when compared to patients with F0–F1 fibrosis [19,424.6 (13,232.1–24,783.0 ng/mL) vs. 14,733.9 (10,789.0–19,822.3 ng/mL), *p* = 0.014].

Plasma I-FABP was significantly correlated with LBP (r = 0.368, *p* < 0.001) and MRE (r = 0.356, *p* < 0.001), as well as the abundance of *Parabacteroides* (r = 0.252, *p* = 0.007) and *Escherichia_Shigella* (r = 0.258, *p* = 0.006). The plasma I-FABP level also showed a negative correlation with the abundance of *Lachnospira* (r = −0.214, *p* = 0.023) but did not correlate with other bacterial genera and MRI-PDFF. For plasma LBP, this marker showed a significant correlation with MRE (r = 0.218, *p* = 0.006) but did not correlate with the MRI-PDFF value or the abundance of bacterial genera.

To compare the levels of circulating inflammatory cytokine between groups, serum IL-6 and TNF-α were assessed. Patients with F2–F4 had higher levels of IL-6 than those with F0–F1 fibrosis, but these were not significant (46.5 ± 69.9 vs. 19.8 ± 18.4 pg/mL, *p* = 0.097). A similar trend was also observed in serum TNF-α concentrations (30.5 ± 38.5 vs. 17.1 ± 15.1 pg/mL, *p* = 0.113). IL-6 concentrations were strongly correlated with serum TNF-α levels (r = 0.883, *p* < 0.001). Serum IL-6 also correlated with serum AST (r = 0.264, *p* = 0.004) and ALT (r = 0.240, *p* = 0.009), while serum TNF-α had a positive correlation with ALT level (r = 0.206, *p* = 0.023). However, these inflammatory cytokines did not show any significant correlation with other clinical parameters and the abundance of bacterial genera.

### 3.7. Distributions of SNPs according to Fibrosis Stages

The genotype frequencies of *PNPLA3* rs738409, *TM6SF2* rs58542926, and *HSD17B13* rs6834314 did not deviate from the Hardy–Weinberg Equilibrium (*p* > 0.05). In the whole cohort, the distributions of *PNPLA3* (CC/CG/GG) were 43 (27.6%)/63 (40.4%)/50 (32.1%), while the frequencies of *TM6SF2* (CC/CT/TT) were 124 (79.5%)/27 (17.3%)/3 (1.9%), and the frequencies of *HSD17B13* (AA/AG/GG) were 64 (41.0%)/72 (46.2%)/18 (11.5%). Patients with F2–F4 fibrosis had an increased frequency of the PNPLA3 GG genotype in comparison to those in the F0–F1 group [odds ratio (OR) = 2.75; 95% confidence intervals (CI): 1.15–6.58; *p* = 0.023]. Interestingly, when taking the combination of gender and PNPLA3 into consideration, our data showed that female patients harboring the GG genotype had a significantly higher percentage of F2–F4 than those with the CC/CG genotypes (36.8 vs. 12.3%, *p* = 0.035). However, this observation was not found among male individuals (Figure S3, Supplementary Materials).

When connected to gut dysbiosis, patients harboring the *PNPLA3 GG genotype* had a significantly lower F/B ratio than those with PNPLA3 CC + CG genotypes (2.0 ± 1.3 vs. 2.7 ± 2.3, *p* = 0.015). However, there was no significant association between *PNPLA3* polymorphisms and the relative abundance of bacterial genera. In this report, the frequencies of *TM6SF2* CT + TT in the F2–F4 group were comparable to the F0–F1 group (OR = 0.76; 95%CI: 0.24–2.39; *p* = 0.632). Similarly, the distributions of HSD17B13 AG + GG were not different between the studied groups (OR = 1.08; 95%CI: 0.45–2.59; *p* = 0.863).

### 3.8. Univariable and Multivariable Analyses

Based on binary logistic regression, we further investigated whether the studied parameters were independently associated with more advanced fibrosis (F2–F4). The variables entered into univariable and multivariable analyses included age, gender, BMI, T2DM, HT, DLP, AST, ALT, platelet count, liver steatosis, SNPs, F/B ratio, gut microbial genera, and dietary patterns. Multivariable analysis indicated that older age, the presence of T2DM, high AST, PNPLA3 rs738409 GG genotype and specific gut microbial genera (enriched *Escherichia_Shigella* and *Fusobacterium*, and depleted *Lachnospira*) were independently associated with moderate/severe fibrosis in patients with MASLD (Table 2).

### 3.9. Random Forest Classification

**Figure 5:**
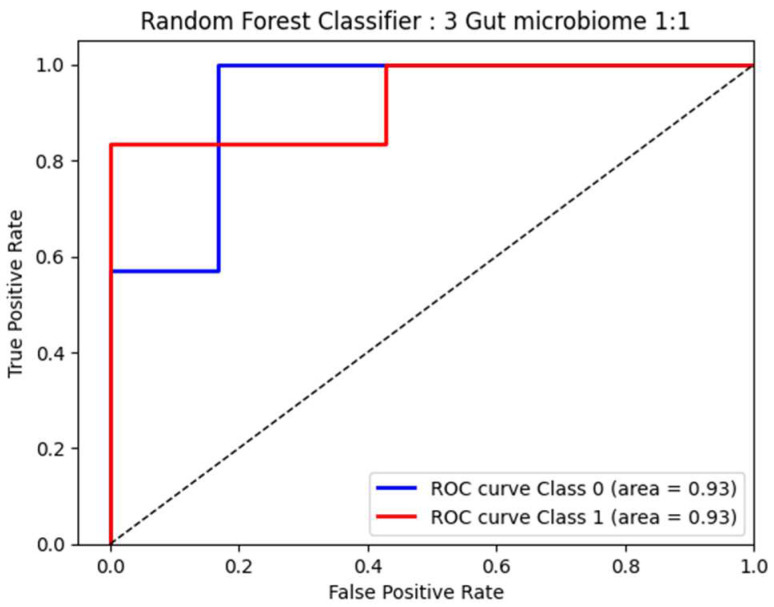
ROC curve analysis based on 3 significant bacteria by Random Forest.

To explore the performance of bacterial abundance in classifying patients with F0–F1 and F2–F4 fibrosis (at a 1:1 ratio) who were age- and sex-matched, we selected three bacteria, including *Escherichia_Shigella*, *Fusobacterium*, and *Lachnospira*, which are independent factors related to F2–F4 fibrosis, to perform the Random Forest classification. Following this method, our result showed a high area under the ROC curve (AUC) at 0.93 (95% CI: 0.53–1.32; *p* = 0.980) for the discrimination between F0–F1 and F2–F4 (Figure 5). These data suggested that these bacterial genera exhibited great diagnostic performance in differentiating between moderate/severe fibrosis and no/mild fibrosis in patients with MASLD.

## 4. Discussion

MASLD represents one of the most common liver diseases worldwide and its prevalence continues to increase gradually. MASLD is now considered a multifactorial disease involving several risk factors including age, the presence of metabolic disorders, and host genetic variants. In this study, we demonstrated that old age, T2DM, high AST level, and PNPLA3 variants were significantly associated with advanced fibrosis stages (F2–F4), which were in agreement with most reports in the literature [3,20]. In addition, several data reports investigated the association between gut dysbiosis and disease severity of MASLD. Thus, one of the challenges in considering the actual connection between gut microbiota and MASLD is to combine other important co-variables into account for the statistical analysis. Based on multivariable analysis, our data showed that distinct alterations in gut microbiota were independently associated with significant/advanced fibrosis. Moreover, the combination of the bacterial genera as a gut microbial signature was able to differentiate F2–F4 vs. F0–F1 with high diagnostic accuracy. These results might indicate that gut dysbiosis is distinct according to the disease severity, which provides evidence supporting the role of gut microbiota in the pathogenesis and progression of MASLD.

Gut dysbiosis has been shown to be associated with the development and progression of MASLD [11]. In this report, we observed significant changes in gut microbiota diversity and composition between individuals according to the fibrosis severity of MASLD. Regarding alpha-diversity, there was no significant difference in Chao1, Shannon, and Simpson indices between patients with F0–F1 and F2–F4, indicating that the community richness and evenness might not be related to disease severity. However, beta diversity significantly differed between the two groups, suggesting variability in the clustering of gut microbiota composition was linked to fibrosis stages. At the phylum level, it is generally recognized that Firmicutes and Bacteroidetes are the most common intestinal bacteria, accounting for more than 90% of the whole microbial populations [21], and thus the F/B ratio could reflect gut homeostasis. In our cohort, we observed a trend of decreased F/B ratios in patients with more advanced disease versus those with less severe fibrosis. This finding was partly in agreement with the observations reported in most studies demonstrating a significant decline in the F/B ratio in advanced MASLD [22]. However, it should be mentioned that the F/B ratio may vary among different cohorts and could yield inconsistent results depending on several factors, such as different molecular techniques (i.e., 16S rRNA vs. shotgun metagenome sequencing) and BMI of the studied populations (i.e., obese vs. non-obese) [23]. For example, a recent systematic review reveals gut microbiota profiles in adults with obesity and indicates that an increased F/B ratio might be a biomarker of obesity susceptibility [24].

At the genus level, we identified *Fusobacterium* and *Escherichia_Shigella* independently associated with the severity of fibrosis in patients with MASLD. Interestingly, the abundance of *Fusobacterium* exhibited a positive correlation with the values of MRE measurement. *Fusobacterium* is a Gram-negative, opportunistic anaerobic pathogen that acts as a potent stimulator of several cytokines and participates in chronic inflammatory processes, as well as carcinogenesis [25]. For instance, the bacterium is enriched in the colonic tissue of patients with colorectal cancer (CRC) and has been strongly implicated in the multi-step processes of CRC development [26]. *Fusobacterium* was also reported to be predominant in patients with steatohepatitis when compared to individuals with simple steatosis, suggesting its contributing role in the progression of MASLD [27,28]. Regarding *Escherichia_Shigella*, this genus is an ethanol-producing bacterium that can lead to de novo lipogenesis and decrease fatty acid oxidation, both of which could induce the occurrence of liver steatosis [29]. Moreover, ethanol and other endotoxins, such as lipopolysaccharides (LPSs), generate high levels of reactive oxygen species, resulting in liver inflammation and the development of fibrosis [30]. It was demonstrated that elevated ethanol levels were detected in patients with steatohepatitis, suggesting that the high abundance of *Escherichia_Shigella* could accelerate progressive MASLD [30,31]. Similar to our report, a previous study also confirmed a greater abundance of *Escherichia_Shigella* in patients with advanced fibrosis (F2–F4) compared to those with F0–F1 fibrosis [31]. Likewise, *Escherichia_Shigella* was shown to be significantly related to necro-inflammatory activity and fibrosis in the liver biopsy of morbidly obese patients with MASLD [32]. A recent meta-analysis also confirmed the enrichment of *Fusobacterium* and *Escherichia* involved in progressive liver disease through increased pro-inflammatory states in advanced MASLD [33].

Short-chain fatty acids (SCFAs) are key mediators that modulate several physiological functions of the intestinal epithelium, including cell proliferation, energy regulation, barrier integrity, and anti-inflammatory process [34]. SCFAs also play an essential role in modifying disease progression in MASLD by the regulation of intrahepatic fat and glucose metabolism, as well as inflammatory response [35]. In animal and human studies of MASLD, increasing SCFA-producing bacteria and fecal SCFA concentrations have been shown to protect against liver injury and steatosis [36]. In this study, *Lachnospira*, a SCFA-producing bacterium, was found to be significantly diminished in the F2–F4 group compared to the less severe group. Of note, the bacterial abundance in fecal samples was also negatively correlated with liver stiffness values measured by MRE. It was shown that a decreased abundance of *Lachnospira* was associated with an increased risk of cardiometabolic disorders, including overweight/obesity, T2DM, MASLD, and CVD [34,37,38]. Interestingly, a recent cross-sectional study in China showed that the relative abundance of *Lachnospira* declined gradually from early to late-stage alcoholic-associated liver disease (ALD), while *Fusobacterium* and *Escherichia_Shigella* were significantly enriched as the disease advanced [39], which was remarkably similar to our results. Thus, it appears that the progressive imbalance of gut microbial composition, i.e., the enrichment of pathogenic bacteria and the reduction in beneficial bacteria, occurs in parallel with the development of more advanced MASLD and ALD. Interestingly, our data based on the Random Forest classifier model revealed that the gut microbial signatures could discriminate the F2–F4 group from the F0–F1 group with high diagnostic accuracy (AUC of 0.93). In this context, our data confirm previous reports that the identification of microbial signatures has a strong predictive value for significant fibrosis to cirrhosis in MASLD [40,41,42,43]. Indeed, a microbiota-based approach could open opportunities for potential utility to improve clinical outcomes as the current options for prevention and therapy for patients with MASLD are restricted.

Current data indicate that gut dysbiosis is linked to an altered intestinal barrier and increased microbial translocation from the intestinal lumen into the liver via the gut–liver axis. Translocated bacterial components and metabolites, such as LPS and other endotoxins, could, in turn, activate the immune response and induce liver inflammation that potentially drives MASLD progression [10]. In this study, we further verified the mechanisms of gut dysbiosis involving fibrosis progression by determining the circulating I-FABP and LBP levels, the surrogate markers of intestinal permeability, and microbial translocation, respectively [44]. Our results demonstrated that the two markers were significantly increased in patients with F2–F4 compared with those with F0–F1 fibrosis. Moreover, the levels of I-FABP and LBP had a positive correlation with the abundance of *Escherichia_Shigella*. Recent evidence from a meta-analysis has also shown that inflammatory cytokines, including IL-6 and TNF-α among others, are significant factors contributing to the development and progression of MASLD [45]. In this study, we demonstrated that serum IL-6 and TNF-α levels tended to be elevated in the F2–F4 fibrosis vs. the F0–F1 group, although a significant difference was not reached. These data indicated that altered gut barrier function combined with enhanced microbial translocation paralleled a rise in the severity of liver fibrosis in patients with MASLD. In line with our data, a recent meta-analysis has demonstrated that increased circulating endotoxin levels are detected in patients with MASLD with advanced fibrosis compared to those with mild fibrosis, underlining the impact of intestinal barrier disruption and endotoxins in disease progression [46].

The characterization of the possible functionality of gut microbiota in the pathogenesis of MASLD was further performed. Based on the functional analysis using PICRUSt2, our data demonstrated that the enriched pathways, according to fibrosis severity, were mostly involved in the biosynthesis of various substrates, including amino acids, nucleosides, nucleotides, and vitamins. Among them, L-glutamate and L-glutamine biosynthesis was one of the most distinct pathways predominately found in the F0–F1 group vs. the F2–F4 group. This pathway generates a complex mixture of metabolic end products, including SCFAs, that influence gut epithelial functions and modulate mucosal immunity [47]. Glutamine could also impact gut microbial communities through diverse mechanisms, including the activation of NF-κB and PI3K-Akt pathways, decreasing bacterial overgrowth or translocation, and increasing the production of secretory immunoglobulin A against pathogens [48]. Recently published data in an animal model also demonstrated that glutamine was able to ameliorate liver steatosis via the regulation of glycolipid and gut microbiota [49]. On the contrary, an additional pathway that predominated in the F2–F4 groups was L-lysine degradation. It was revealed that distinct gut microbiota in pediatric MASLD was related to more alcohol production and enhanced degradation of various amino acids, including lysine, a branched-chain amino acid [50]. Likewise, a recent study in patients with MASLD also showed that increased lysine and histidine degradation in fecal content could be microbial biomarkers for high-grade liver steatosis [51].

It is well documented that dietary patterns and nutrient compositions have significant impacts in promoting or counteracting the development and progression of MASLD [52]. Moreover, the heterogeneity of gut microbial composition can be modulated positively or negatively by different dietary patterns [13]. Thus, complex interactions between dietary factors and gut microbiota could affect human metabolic health and disease, including MASLD. However, current data regarding the relationship between dietary patterns and gut microbiota in terms of disease severity in patients with MASLD are inadequate. In our study, the dietary patterns (vegetable-, meat-, and carbohydrate-based diet) were not directly associated with fibrosis severity. However, a vegetable-based diet had a trend toward increased abundance of *Lachnospira*, compared to high meat or carbohydrate consumption. It has been shown that fiber-rich diets could improve gut microbial composition and function, as well as increase SCFA-producing bacteria and SCFA concentrations, which promote cardiometabolic health [34]. Regarding the impact of high protein consumption on MASLD, little is known, as most reports focus on carbohydrates and fats. Moreover, the role of a meat-based diet in MASLD development remains controversial, depending on the type and amount of protein intake [52]. Despite this inconclusive data, an unhealthy Western diet with a high protein intake is thought to be related to the increased prevalence and severity of MASLD [53]. Interestingly, a recent prospective study also demonstrated that high meat consumption was significantly associated with approximately 2.6-fold steatosis and 4.8-fold significant fibrosis compared to consistently low protein intake [54]. Additionally, it was shown that a high protein diet could induce intestinal inflammation by increasing potentially detrimental gut microbiota (e.g., genera *Enterococcus*, *Streptococcus*, and *Escherichia*) [55]. In this regard, although our cross-sectional study did not show a direct relationship between high meat intake and advanced fibrotic stages, the results could indirectly link the disease severity to a meat-based diet as enriched bacterial genera, including *Fusobacterium* and *Escherichia_Shigella,* were found to be associated with advanced fibrosis. Together, it might imply from our data that the consumption of specific dietary patterns could positively or negatively impact the disease severity of patients with MASLD.

Genetic factors have been consistently shown to play a role in the pathogenesis of MASLD [9]. In this study, we also evaluated the impact of genetic variants in *PNPLA3*, *TM6SF2*, and *HSD17B13* on the severity of fibrosis in patients with MASLD. We found that the *PNPLA3* rs738409 GG genotype exhibited a significantly greater distribution in the F2–F4 group versus the F0–F1 group. In the multivariable analysis, the GG genotype remained significant in predicting moderate/severe fibrosis. Our result was in line with most reports demonstrating an increased risk of this polymorphism in patients with advanced MASLD and ALD [56]. Moreover, female patients with the *PNPLA3* rs738409 GG genotype displayed more severe fibrosis compared to those with the CC/CG genotypes, supporting the notion of *PNPLA3* and female gender interaction in disease susceptibility and progression in MASLD [57]. Notably, our results also revealed that the GG genotype commonly displayed a lower F/B ratio when compared to the CC + CG genotypes but did not have any correlation with the bacterial genera. *PNPLA3* polymorphism is by far the most common genetic determinant of MASLD and has potentially been used as part of a personalized management strategy. Indeed, the *PNPLA3* protein is mostly expressed in the liver and displays lipase activity against triglycerides, while Ile148Met substitution leads to a loss of function, leading to triglyceride retention in the hepatocytes [58]. Additionally, it has been demonstrated that its function is required for hepatic stellate cell (HSC) activation, and the variant could confer proinflammatory and profibrogenic features to HSCs [59]. In this study, our cohort did not confirm the role of *TM6SF2* and *HSD17B13* polymorphisms in association with fibrosis stages.

We acknowledged some limitations in our study. First, this study mostly established the associations between gut microbiota and clinical variables, which might not be able to prove a cause-and-effect relationship. Additionally, there were several weak but statistically significant correlations between the variables observed in this study. These findings might indicate the complexity and heterogeneity of gut microbiota influenced by various factors, including diet, lifestyle, host genetics, and environment associated with MASLD. Furthermore, this report was a cross-sectional study in a single institute, and the sample size of patients with F2–F4 was relatively small, which might reflect the lower distribution of more advanced fibrosis/cirrhosis in real-life clinical settings. Thus, prospective longitudinal cohorts with a larger sample size, particularly patients with F2–F4, are necessary to validate our findings. Despite these limitations, our strength was a well-characterized cohort of patients with various disease stages of MASLD. In addition, our data combined several important variables taken into consideration using multiple logistic regression analysis to decrease the confounding effects. Finally, the utility of MRE, which is as a continuous measurement of liver fibrosis, could allow us to determine fibrosis severity in association with other quantitative variables (i.e., positive or negative correlation with the abundance of bacterial genera).

## 5. Conclusions

Our findings demonstrated that several clinical features, including old age, the presence of T2DM, high serum AST, and *PNPLA3* polymorphism, as well as gut dysbiosis, were significantly associated with fibrosis severity in patients with MASLD. The altered gut microbiota signature was characterized by a high abundance of pathogenic bacteria, including *Fusobacterium* and *Escherichia-Shigella*, and the depletion of SCFA-producing genera such as *Lachnospira*. These results emphasize the multifactorial participation in the development and progression of MASLD. Thus, integrative information may help us to obtain a better understanding of the pathogenesis of MASLD, which in turn could provide an approach towards individualized prevention and treatment for patients with MASLD.

## Figures and Tables

**Table 1:** Characteristics of patients in this study.

Characteristics	MASLD (F0–F1) (n = 131)	MASLD (F2–F4) (n = 25)	*p*-Value
Age (years)	54.4 ± 13.5	63.5 ± 10.4	0.002 *
Gender			0.514
Male	69 (57.7)	11 (44.0)	
Female	62 (47.3)	16 (56.0)	
Body mass index (kg/m^2^)			0.751
<23.0	6 (4.6)	2 (8.0)	
23.0–29.9	89 (67.9)	7 (68.0)	
>30.0	36 (27.5)	6 (24.0)	
Presence of type 2 diabetes	33 (25.2)	17 (68.0)	<0.001 *
Presence of hypertension	48 (36.6)	16 (64.0)	0.014 *
Presence of dyslipidemia	50 (38.2)	8 (32.0)	0.655
Total cholesterol (mg/dL)	187.9 ± 36.0	181.4 ± 43.9	0.455
HDL cholesterol (mg/dL)	49.4 ± 12.1	53.8 ± 15.7	0.144
LDL cholesterol (mg/dL)	121.2 ± 35.1	111.2 ± 40.2	0.245
Triglyceride (mg/dL)	131.4 ± 55.6	144.7 ± 62.8	0.315
Hemoglobin (g/dL)	14.0 ± 1.9	13.2 ± 1.5	0.160
White blood count (10^3^/µL)	6.9 ± 2.0	7.0 ± 2.2	0.931
Platelet count (10^3^/µL)	264.3 ± 63.8	196.8 ± 69.0	<0.001 *
Total bilirubin (mg/dL)	0.7 ± 0.3	0.7 ± 0.3	0.983
Serum albumin (g/dL)	4.4 ± 0.2	43.2 ± 21.5	0.110
Aspartate aminotransferase (IU/L)	25.5 ± 10.3	43.2 ± 21.5	<0.001 *
Alanine aminotransferase (IU/L)	36.5 ± 22.6	47.7 ± 24.7	0.026 *
Alkaline phosphatase (IU/L)	72.3 ± 19.4	82.3 ± 12.5	0.139
Magnetic resonance elastography (kPa)	2.3 ± 0.3	4.5 ± 1.3	<0.001 *
Proton density fat fraction (%)	14.0 ± 7.5	11.2 ± 6.0	0.077

Data expressed as mean ± SD or n (%); * *p*-value < 0.05.

**Table 2:** Factors associated with significant fibrosis to cirrhosis (F2–F4).

Factors	Category	Univariable Analysis		Multivariable Analysis	
		OR (95%CI)	*p*-Value	OR (95%CI)	*p*-Value
Age (years)	≥55 vs. <55	4.06 (1.44–11.47)	0.008 *	6.30 (1.18–33.76)	0.032 *
Gender	Male vs. Female	0.71 (0.30–1.67)	0.428		
BMI (kg/m^2^)	≥25 vs. <25	0.98 (0.36–2.68)	0.971		
Diabetes	Yes vs. No	6.31 (2.49–15.97)	<0.001 *	5.11 (1.42–18.44)	0.013 *
Hypertension	Yes vs. No	3.07 (1.26–7.49)	0.013 *	3.32 (0.91–12.09)	0.069
Dyslipidemia	Yes vs. No	0.76 (0.31–1.90)	0.559		
Aspartate aminotransferase (IU/L)	≥40 vs. <40	2.80 (1.01–7.73)	0.048 *	10.92 (2.13–56.09)	0.004 *
Alanine aminotransferase (IU/L)	≥40 vs. <40	2.30 (0.97–5.46)	0.060		
Platelet count (10^9^/L)	<150 vs. ≥150	7.50 (1.95–28.78)	0.003 *	6.78 (1.00–45.98)	0.050
Liver steatosis grade	S2 + S3 vs. S1	2.02 (0.76–5.39)	0.161		
*PNPLA3* rs738409	GG vs. CC + CG	2.75 (1.15–6.58)	0.023 *	9.50 (2.23–40.50)	0.002 *
*TM6SF2* rs58542926	CT + TT vs. CC	0.76 (0.24–2.39)	0.632		
*HSD17B13* rs6834314	AA vs. AG + GG	1.08 (0.24–2.39)	0.863		
*Firmicutes/Bacteroidetes* ratio	High vs. Low	0.71 (0.46–1.09)	0.121		
*Lachnospira*	Low vs. High	3.04 (1.19–7.78)	0.020 *	5.11 (1.24–21.11)	0.024 *
*Parabacteroides*	High vs. low	3.71 (0.59–23.44)	0.163		
*Escherichia-Shigella*	High vs. low	4.71 (1.59–13.91)	0.005 *	6.11 (1.12–33.19)	0.036 *
*Fusobacterium*	High vs. low	2.95 (1.16–7.54)	0.024 *	8.66 (1.79–41.98)	0.007 *
*Bacteroides*	High vs. low	1.98 (0.82–4.80)	0.131		
Dietary patterns	D2 + D3 vs. D1	1.60 (0.43–5.99)	0.486		

Data expressed as odds ratio (OR) and 95% confidence intervals (CI); D1 = vegetable-based diet; D2 = meat-based diet; D3 = carbohydrate-based diet; * *p*-value < 0.05.

## Data Availability

Data are available in the Sequence Read Archive (SRA), under submission number SUB14322992 (BioProject ID: PRJNA1089406).

## Supplementary Materials

The following supporting information can be downloaded at: https://www.mdpi.com/article/10.3390/nu16121800/s1. Table S1: Characteristics of patients according to gender: (a) female; (b) male. Figure S1: Top 50 relative bacterial compositions at the genus level in the F0–F1 and F2–F4 groups. Figure S2: Relative abundance of bacterial genera according to dietary patterns. Figure S3: The combination analysis of the sex and genotype of patients with PNPLA3.
