# Supplementary material for: Distinct Gut Microbial Signature and Host Genetic Variants in Association with Liver Fibrosis Severity in Patients with MASLD
Source: Nutrients. 2024 Jun 7;16(12):1800. doi: 10.3390/nu16121800 (PMC11206871; doi:10.3390/nu16121800)
Supplement: Supplementary file 1 [file nutrients-16-01800-s001.zip › nutrients-2948865-supplementary.pdf]

Supplementary Table S1. Characteristics of patients according to gender: (a) female; (b) male.

(a)

Characteristics	MASLD (F0-F1) (n=62)	MASLD (F2-F4) (n=14)	*p*-value
Age (years)	58.6±13.5	64.4±8.9	0.128
Body mass index (kg/m^2)			
<23.0	4(6.5)	1(7.1)	0.960
23.0-29.9	38(61.2)	8(57.2)	
>30.0	20(32.3)	5(35.7)	
Presence of type 2 diabetes	18(29.0)	10(71.4)	0.005*
Presence of hypertension	24(38.7)	8(57.1)	0.241
Presence of dyslipidemia	24(38.7)	6(42.9)	0.772
Hemoglobin (g/dL)	13.0±1.4	12.8±1.3	0.650
White blood count (10^3/μL)	6.9±2.6	7.0±2.2	0.992
Platelet count (10^3/μL)	269.8±64.4	197.3±90.0	0.001*
Total bilirubin (mg/dL)	0.7±0.3	0.8±0.3	0.429
Serum albumin (g/dL)	4.3±0.2	4.3±0.3	0.969
Aspartate aminotransferase (IU/L)	25.3±11.7	48.7±24.0	0.003*
Alanine aminotransferase (IU/L)	31.8±18.3	50.9±27.3	0.002*
Alkaline phosphatase (IU/L)	73.7±23.7	82.9±9.6	0.149
Magnetic resonance elastography (kPa)	2.3±0.4	4.7±1.4	<0.001*
Proton density fat fraction (%)	13.4±7.6	12.2±7.0	0.595

(b)

Characteristics	MASLD (F0-F1) (n=69)	MASLD (F2-F4) (n=11)	*p*-value
Age (years)	50.7±12.4	62.4±12.5	0.005*
Body mass index (kg/m^2)			
<23.0	2(2.9)	1(9.1)	0.380
23.0-29.9	51(73.9)	9(81.8)	
>30.0	16(23.2)	1(9.1)	
Presence of type 2 diabetes	15(21.7)	17(68.0)	0.008*
Presence of hypertension	24(34.8)	8(72.7)	0.023*
Presence of dyslipidemia	26(37.7)	2(18.2)	0.312
Hemoglobin (g/dL)	14.7±1.9	13.8±1.7	0.235
White blood count (10^3/μL)	6.9±1.5	7.0±2.3	0.989
Platelet count (10^3/μL)	258.9±63.2	196.1±28.0	<0.001*
Total bilirubin (mg/dL)	0.8±0.3	0.6±0.3	0.285
Serum albumin (g/dL)	4.4±0.2	4.2±0.2	0.117
Aspartate aminotransferase (IU/L)	25.7±8.9	36.2±16.4	0.002*
Alanine aminotransferase (IU/L)	40.7±25.2	43.6±21.7	0.714
Alkaline phosphatase (IU/L)	71.3±15.9	80.5±26.2	0.443
Magnetic resonance elastography (kPa)	2.3±0.3	4.3±1.2	<0.001*
Proton density fat fraction (%)	14.6±7.4	9.9±4.4	0.047*

Data are expressed as mean ± SD or n (%); *p-value <0.05.

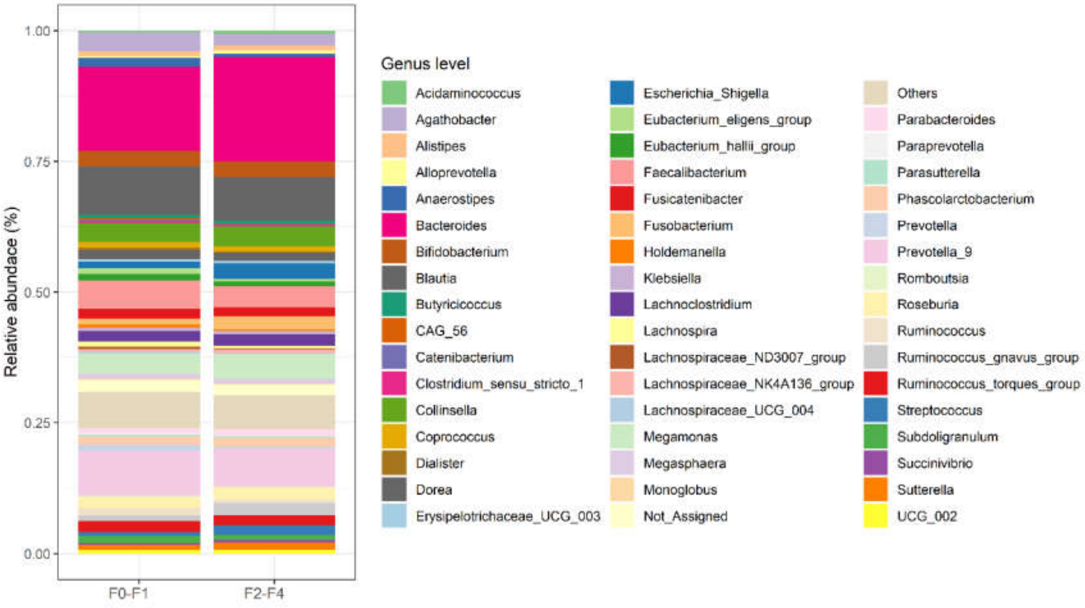

Figure S1. Top 50 relative bacterial compositions at the genus level in the F0–F1 and F2–F4 groups.

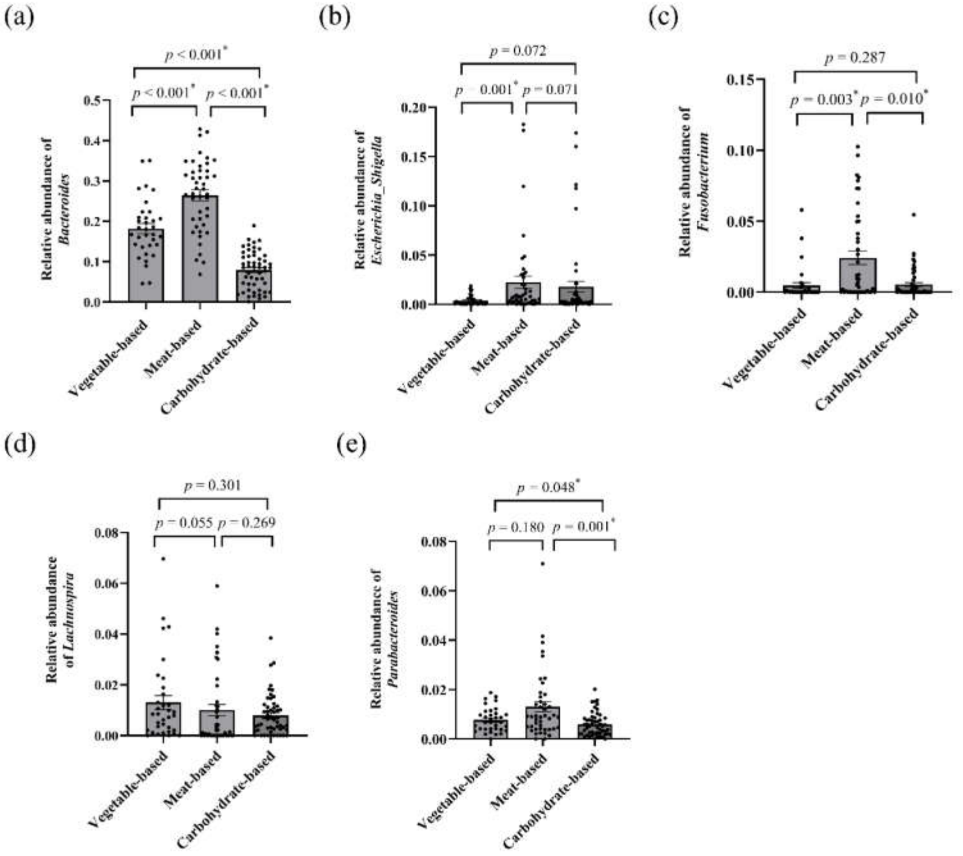

Figure S2. Relative abundance of bacterial genera according to dietary patterns.

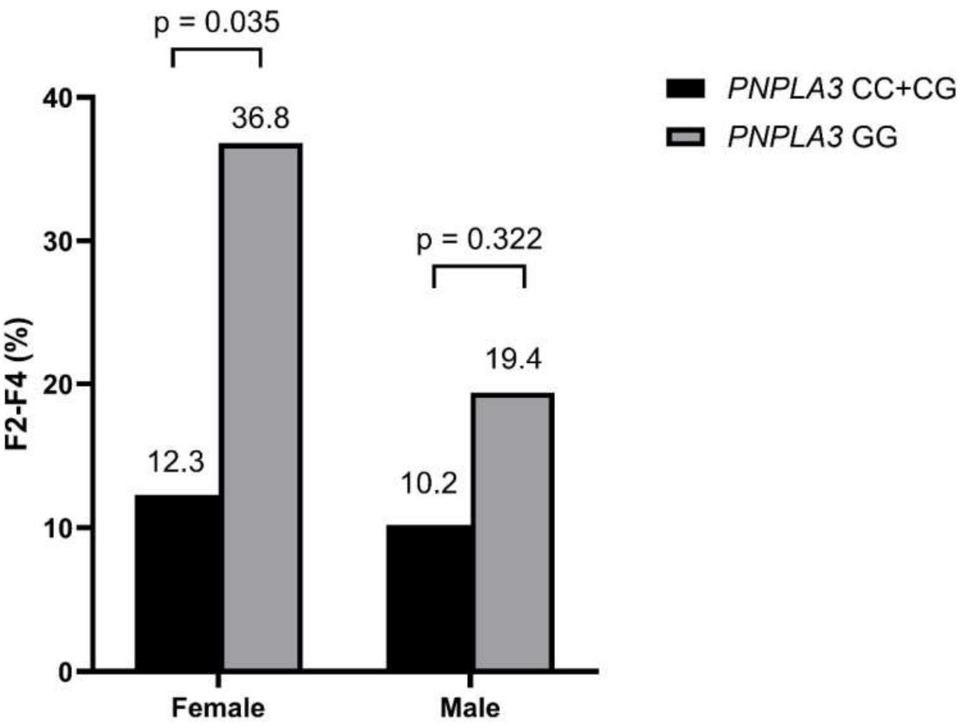

Figure S3. The combination analysis of the sex and genotype of patients with PNPLA3.
